# Disparities in excess deaths from the COVID-19 pandemic among migrant workers in Kuwait

**DOI:** 10.1186/s12889-021-11693-w

**Published:** 2021-09-14

**Authors:** Barrak Alahmad, Dawoud AlMekhled, Ayah Odeh, Dalia Albloushi, Janvier Gasana

**Affiliations:** 1 Department of Environmental and Occupational Health, Faculty of Public Health, Kuwait University, Kuwait City, Kuwait; 2 Environmental Health Department, Harvard T.H. Chan School of Public Health, Harvard University, Boston, MA USA; 3 School of Biomedical Sciences, Faculty of Medicine, Nursing, and Health Sciences, Monash University, Melbourne, Australia; 4 Department of Health Policy and Management, Faculty of Public Health, Kuwait University, Kuwait City, Kuwait; 5 Mubarak Al-Kabeer Hospital, Ministry of Health, Hawalli, Kuwait

**Keywords:** Migrant workers, Excess deaths, Health disparities, Kuwait, COVID-19, Pandemic

## Abstract

**Background:**

The actual human cost of the pandemic cannot be viewed through the COVID-19 mortality rates alone, especially when the pandemic is widening the existing health disparities among different subpopulations within the same society. In Kuwait, migrant workers were already disproportionately impacted by COVID-19 and its unintended consequences. The totality of that effect on mortality is yet to be fully understood.

**Objective:**

To estimate excess deaths in the pandemic year of 2020 among the Kuwaiti and non-Kuwaiti migrant populations.

**Methods:**

We analyzed publicly available retrospective data in Kuwait on total annual mortality historically (from 2005 to 2019) and in 2020. We fitted a quasi-poisson generalized linear model adjusted for yearly trend and nationality to estimate the expected deaths in 2020 in the absence of the pandemic. We calculated excess deaths as the difference between observed and expected mortality for the year of the pandemic in both Kuwaitis and non-Kuwaitis.

**Results:**

In the absence of the pandemic, we expected the total mortality in Kuwait to be 6629 (95% CI: 6472 to 6789) deaths. However, the observed total mortality in 2020 was 9975 deaths; about 3346 (3186 to 3503) more deaths above the expected historical trend. Deaths among migrant workers would have been approximately 71.9% (67.8 to 76.0) lower in the absence of the pandemic. On the other hand, deaths among Kuwaitis would have been 32.4% (29.3 to 35.6) lower if the country had not been hit by the pandemic.

**Conclusion:**

The burden of mortality brought on by the COVID-19 pandemic is substantially higher than what the official tally might suggest. Systematically disadvantaged migrant workers shouldered a larger burden of deaths in the pandemic year. Public health interventions must consider structural and societal determinants that give rise to the health disparities seen among migrant workers.

**Supplementary Information:**

The online version contains supplementary material available at 10.1186/s12889-021-11693-w.

## Introduction

The coronavirus disease 2019 (COVID-19) pandemic triggered a mass disruption to social, economic, and environmental modes of operation. Most alarming is the disruption to the health system caused by the pandemic, that will manifest into the overall population health [1]. Governments worldwide have put efforts to mitigate the burden of the pandemic on the healthcare system by reducing access to healthcare in non-urgent circumstances; through cutting down routine testing of other diseases, cancellations of elective surgeries and in-person consultations with patients with chronic diseases [1, 2]. The COVID-19 mortality rates alone do not indicate the total burden of disease during the pandemic year as disruptions to healthcare access are likely to result in greater mortality. Furthermore, due to differences in death reporting protocols, relying on COVID-19 mortality rates as a comparator is not a reliable way to measure the effects of the pandemic between countries [3].

In order to assess the overall impact of the pandemic, excess death must be carefully examined [3, 4]. Excess death is the difference between the number of deaths from all causes observed during a public health crisis and the expected number of deaths in its absence or in otherwise ‘normal’ conditions. This provides an efficient mortality surveillance strategy accounting for the indirect burden of disease caused by disruptions to the access and use of healthcare [4]. This estimate overcomes the variability between different countries in how they report COVID-19 deaths as well as allowing for a clearer indication of the impact of the pandemic in each country. Excess deaths have been previously used to assess the human costs of prior pandemics and to estimate the impact of climate change on mortality [5–9].

Ethnic and racial disparities have existed in healthcare access and utilization prior to the COVID-19 pandemic [1, 10]. In the US alone, the Black community has experienced lower standards of health, lower life expectancies, and higher mortalities compared to their white counterparts [11, 12]. Similarly, in Kuwait, migrant worker communities’ differential access to education, housing, healthcare and employment reduces their quality of overall health. Migrant workers who make up the majority of the population are employed in low skilled labour jobs and domestic work, facing precarious working conditions, financial hardships and inadequate housing [13]. Having little knowledge of the health insurance systems and language barriers makes it difficult for them to access healthcare when needed. Additionally, migrants are more likely to work in occupations that increase the risk of transmission and are generally excluded from protections in public policies [13]. All these factors contribute to a structural and systematic health disadvantage that gives rise to health disparities in Kuwait amongst ethnic populations.

Kuwait has been severely affected by COVID-19. Due to the imposition of lockdowns and curfews to mitigate transmission rates, the country has witnessed a disruption to healthcare access, delivery, and provision. Kuwait has a population of 4.7 million, with a demographic profile consisting of approximately two-thirds non-Kuwaiti [14]. Previous studies in Kuwait have highlighted the disparities in COVID-19 exposure risk and adverse health outcomes [13, 15–17]. However, to date there has been no study assessing the excess deaths in Kuwait as well as between Kuwaitis and non-Kuwaitis attributable to the COVID-19 pandemic. This study aims to estimate the excess deaths in the pandemic year of 2020 among the Kuwaiti and non-Kuwaiti subpopulations.

## Methods

### Data sources

Data was collated from publicly available official sources by the Kuwaiti Government. Annual population estimates by nationality from 2005 to 2020 was obtained from the Public Authority for Civil Information (accessible: https://www.paci.gov.kw/stat/TimeSeries.aspx). Similarly, deaths by nationality in 2005 and 2020 were obtained from the same source. In an additional analysis, we used historical mortality data between 2001 and 2018 by nationality from annual mortality reports prepared by the government’s Central Statistical Bureau (accessible: https://csb.gov.kw/Pages/Statistics?ID=10&ParentCatID=1). The compiled data that supports this analysis is available in the supplemental material (Supplemental Raw Data).

To compare our dataset with other countries, we used the World Mortality Dataset (accessible: https://github.com/akarlinsky/world_mortality) and the COVID-19 excess deaths data from ‘Our World In Data’ – a project of the Global Change Data Lab, a registered charity in England and Wales that is run by Oxford Marin School, University of Oxford (accessible: https://ourworldindata.org/excess-mortality-covid) [18]. The methods used to calculate excess deaths in other countries are described elsewhere [19]. We summed weekly or monthly observed and expected deaths from countries that provided complete datasets.

### Statistical analysis

We calculated an unadjusted death rate as the number of deaths divided by the population estimate per 1000 individuals per year. We used two methods to calculate expected deaths in 2020. First, we fitted a generalized linear regression for rates of annual deaths from 2001 to 2019 with a quasi-poisson distribution to account for overdispersion. We included two independent variables: linear trend for year, and a dummy variable for nationality (Kuwaiti vs. Non-Kuwaiti). Additionally, we included an offset term log (population) to model the rate rather than counts and account for changes in population dynamics over the years. We then used the model to predict the expected 2020 deaths with 95% confidence intervals (CI) for Kuwaitis and non-Kuwaitis. In a sensitivity analysis, to check for non-linearity of yearly trends of mortality rates, we fitted a generalized additive model with penalized splines for years.

Further, we used the simple rolling average method to calculate the expected deaths in 2020. We averaged deaths in the last 5 years (2015 to 2019) for Kuwaitis and non-Kuwaitis. Then, we calculated the uncertainty around the averaging estimate (95% CI) by adding and subtracting the estimate from 1.96 times the square root of the estimate [20].

Excess deaths were calculated as observed deaths minus expected deaths in 2020 for each subpopulation. Percent increase in excess deaths was calculated by subtracting expected from observed deaths and then dividing by expected deaths. A similar approach was carried out for upper and lower bounds of expected deaths from the two methods.

## Results

The number of deaths and death rates per 1000 from 2015 to 2020 are presented in Table 1. Overall, 9975 deaths were reported in 2020, with an overall rate of 2.1 deaths per 1000 population; a marked increase compared to the previous 5 years. Similarly, when examined by nationality, the total number of deaths among Kuwaitis in 2020 was 4756 with a rate of 3.3 deaths per 1000 population, while non-Kuwait deaths totaled to 5219 deaths with a rate of 1.6 per 1000 population.

**Table 1:** Total mortality counts and death rates in Kuwait from 2015 to 2020

Year	Total		Kuwaitis		Non-Kuwaitis	
	Deaths	Rate per 1000	Deaths	Rate per 1000	Deaths	Rate per 1000
2015	6099	1.4	3261	2.5	2838	1.0
2016	6248	1.4	3439	2.6	2809	0.9
2017	6356	1.4	3432	2.5	2924	0.9
2018	6374	1.4	3433	2.4	2941	0.9
2019	6896	1.4	3687	2.6	3209	1.0
2020	9975	2.1	4756	3.3	5219	1.6

Source: Public Authority for Civil Information, Government of Kuwait

**Fig. 1:**
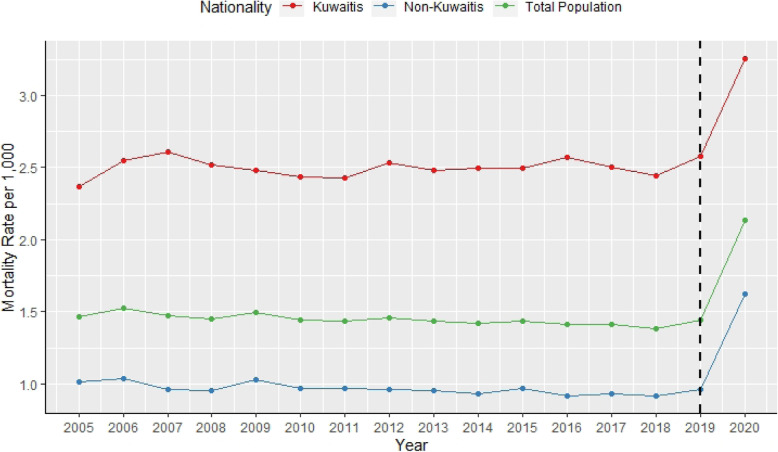
Historical mortality rates in Kuwait per 1000 population from 2005 to 2020

Over the entire historical period of annual mortality examined, there was a general downward trend. The pandemic year of 2020 clearly exhibited a large increase in mortality rate compared to the previous years. This was seen among non-Kuwaitis, increasing from 1.0 to 1.6, and among Kuwaitis as well, having increased from 2.6 to 3.3 deaths per 1000 population (Fig. 1).

Regression analysis showed that expected deaths were significantly lower than observed deaths in 2020, with a total of 3346 (95% CI: 3186 to 3503) excess deaths occurring, a 50.5% (95% CI: 46.9 to 54.1) increase. An estimated 32.4% (95% CI: 29.3 to 35.6) increase in excess deaths was seen among Kuwaitis while 71.9% (95%CI: 67.8 to 76.0) were seen among non-Kuwaitis. Penalized spline on yearly trends did not suggest evidence of non-linearity (degrees of freedom = 1). Simple average estimates showed similar results but slightly higher than the regression analysis, with 3580 (95% CI: 3424 to 3737) more individuals expected dying, accounting for 56.0% increase (95%CI: 52.3 to 59.9) as shown in Table 2. Using the Central Statistical Bureau historical mortality, the results provided a similar pattern, yet the estimates were slightly lower (Supplemental Table A).

**Table 2:** Estimates of expected and excess deaths in 2020 stratified by nationality

	Total	Kuwaitis	Non-Kuwaitis
Observed Deaths in 2020^a^	9975	4756	5219
Regression Estimate:			
*Expected Deaths (95% CI)*	6629 (6472 to 6789)	3592 (3508 to 3678)	3036 (2964 to 3111)
*Excess Deaths (95% CI)*	3346 (3186 to 3503)	1164 (1078 to 1247)	2183 (2108 to 2255)
*Excess Deaths % (95% CI)*	50.5 (46.9 to 54.1)	32.4 (29.3 to 35.6)	71.9 (67.8 to 76.0)
Simple Averaging Estimate:			
*Expected Deaths (95% CI)*	6395 (6238 to 6551)	3450 (3335 to 3566)	2944 (2838 to 3051)
*Excess Deaths (95% CI)*	3580 (3424 to 3737)	1306 (1190 to 1420)	2275 (2168 to 2381)
*Excess Deaths % (95% CI)*	56.0 (52.3 to 59.9)	37.8 (33.9 to 42.6)	77.2 (71.1 to 83.9)

^a^ Source: Public Authority for Civil Information, Government of Kuwait

## Discussion

With the pandemic widening the existing health disparities among different subpopulations within the same society, we observed migrant workers shouldering a larger burden of mortality. There were more than two thousand overall excess deaths amongst non-Kuwaitis translating into more than a 71% increase in expected mortality. In contrast, the Kuwaiti population also experienced a deadly year, yet the percentage increase was nearly 32%, not as dramatic as non-Kuwaitis. That is, nearly a 40-percentage point difference between the two subpopulations in an absolute scale.

Kuwait, like other Gulf Countries, is host to a huge expatriate population and depends heavily on non-Kuwaiti labor. The majority of which are from South and Southeast Asia, mainly India and the Philippines, who are predominately employed in the service industry and construction work, whereas Egyptians compromise the largest of the non-Kuwaiti Arab population [21, 22]. Strict family visa rules led to these workers coming to Kuwait unaccompanied by families who reside in their home countries and depend on their money transfers to make ends meet. The majority of non-Kuwaitis are young males (between 30 and 49 years), and only 20% of non-Kuwaitis have an educational attainment beyond high-school [13]. This imbalance in demographic profiles between the Kuwaiti and non-Kuwaiti population has propelled the state to designate geographical regions as family-only. Hence, isolating the workers from the rest of the population based on gender and nationality in regions that experienced an exponential growth, inevitably straining the infrastructure systems [21]. Moreover, such imbalances in the structure of the Kuwaiti society are bound to have an effect on the distribution of income. The average Kuwaiti income earner earns around KD 1410, while the average non-Kuwaiti income earner earns approximately less than half of that income [23].

Living in cramped and overcrowded homes with poor ventilation while sharing amenities increases the predisposition to COVID-19 infection. In addition, the poor working conditions of migrants could lead to undiagnosed health conditions that when left untreated, may worsen their overall health, leading to severe COVID-19 infections [15]. Even though the basic healthcare needs of migrant workers are covered by national health insurance schemes, access is hindered by limited knowledge of the healthcare system. Moreover, language barriers hamper public health messaging and training on social distancing measures making it difficult for individuals to adhere to COVID-safe practices which increases the risk of infection [13].

The non-Kuwaiti population is comprised of migrant workers who are in working age and hence relatively younger than Kuwaiti adults [14]. The migrant worker subpopulation is generally considered healthier than their counterparts [24]. This can be clearly seen as the historical death rates among the Kuwaiti population was nearly 2.5-fold higher than non-Kuwaitis. However, due to the pandemic alone, despite them being relatively younger, non-Kuwaitis experienced a much higher percentage of excess deaths. This emphasizes that migrant workers are particularly vulnerable to COVID-19 infections and its social, economic and environmental consequences. Migrant workers in Kuwait had more than a two-fold increase in the odds of requiring intensive care or dying from COVID-19 compared to Kuwaitis after adjustment for baseline characteristics including age and co-morbidities [15, 25]. Significant spreading and clustering outbreaks of COVID-19 in Kuwait were shown to be in areas densely populated by migrant workers [16, 17]. A cumulative risk assessment of the effects of COVID-19 on migrant workers in Kuwait showed that stressors arising from domains other than the individual level are inseparable from the risks of adverse health [13]. Migrant workers face job and house insecurity, and are sometimes unable to support their daily needs. Additionally, migrant workers in Kuwait were also shown to be especially vulnerable to death from environmental exposures such as extreme heat, dust storms and air pollution [26, 27]. Disparities in the excess deaths during the pandemic is another confirmatory piece of evidence of structural health inequities that are based on nationality and migrant status in the state of Kuwait.

There are multiple aspects to explain the disparities observed amongst the global excess deaths in a public health crisis. First, the social and environmental determinants of health have been inversely associated with noncommunicable diseases (NCDs); those most deprived are more likely to suffer from NCDs than their more privileged counterparts [28, 29]. Thus, among other things, it is possible that ethnic and racial disparities in COVID-19 mortality and excess deaths are attributable to a higher prevalence of NCDs in marginalized communities. Second, delayed presentation – either due to personal fear of contracting the virus or difficultly accessing the healthcare system, is associated with more extensive disease presentation and therefore requires more aggressive interventions. For instance, a rise in surgical emergencies such as obstructed hernia with bowel ischemia, diabetic foot ulcers requiring amputation and appendicular abscess was noted during this pandemic [30]. Another example is the presentation of complications in oncological patients that have arisen due to the temporary halt of elective surgeries [30]. The same has been observed in medical emergencies, specifically with delayed presentation of myocardial infarction being associated with higher mortality and more complications such as fatal left ventricular free wall rupture and tissue necrosis [31, 32]. Furthermore, the global postponement of medical care most likely resulted in a backlog of appointments, procedures, and surgeries due to increase demand and limited resources. This further delay is likely to have an adverse impact on population health [33].

We compared Kuwait’s excess deaths percentage of 56.0% (simple averaging) to a number of other countries from *Our World In Data* and the *World Mortality Dataset* tracker of excess deaths [18, 19]. The method used to predict expected deaths for other countries in the repository included summing deaths over weeks or months. We were not able to do that for Kuwait given our restricted access to only annual deaths. Yet, at face value, excess deaths in Kuwait were still considerably greater than levels observed in other countries like the U.S. and Europe (Supplemental Figure A). For example, the 2020 annual percentage of excess deaths in the U.S., Spain and Italy are estimated to be 19, 18 and 15%, respectively. Compared to other Gulf states, excess deaths in Kuwait exceeded those from Oman (24%) and Qatar (14%). Comparable numbers from other Gulf countries like Saudi Arabia or the United Arab Emirates were not available. Kuwait’s numbers are however comparable to South American countries such as Ecuador (63%), Bolivia (52%) and Mexico (47%). Given that the pandemic may lead to fewer deaths from accidental causes, the excess numbers of deaths observed in Kuwait in 2020 are concerningly sizeable. It is acknowledged that COVID-19 mortality figures are underestimates of the actual death toll [34, 35]. By December 31st, 2020, the cumulative COVID-19 official reported mortality in Kuwait was 934 deaths [36]. According to our estimates this leaves an additional 2400 deaths unexplained. COVID-19 deaths alone need to be underestimated by a factor of nearly 3.5 to explain the additional mortality. However, we argue that indirect fatalities from delayed and disrupted care due to cancer, circulatory and other causes may have played a significant role in these deaths.

This study has a number of limitations. At the time of writing, important stratifying data on gender, age, cause of death, co-morbidities and year of migration to Kuwait were not available. Secondly, it was not possible for us to accurately account for population decline among migrant workers in the pandemic year due to unemployment or deportation due to invalid paperwork. However, the non-Kuwaiti population estimate from PACI for 2020 showed a noticeable decline which may reflect such changes in population dynamics. This was accounted for in the model as we used log (population) as an offset when estimating the rate of excess deaths. Thirdly, although we present two methods in estimating the expected deaths in 2020, there are more advanced forecasting techniques in predicting expected mortality such as seasonal autoregressive integrated moving average (ARIMA) and machine learning. However, to get accurate estimates using these other methods we would need better temporal resolution (e.g., weekly or monthly mortality data) historically and in 2020. We were limited in obtaining only annual deaths. Daily, weekly, or monthly observed deaths were not publicly available. Because of that, the readers must be cautioned that our overall estimation of excess deaths percentage may be exaggerated. Finally, the disparity in migrant workers excess deaths is restricted to Kuwait and cannot be generalized to other contexts. Yet, the picture is unlikely to be completely different in other Gulf countries that are hosts to millions of migrant workers.

## Conclusion

Based on historical annual deaths in Kuwait, the burden of mortality of the COVID-19 pandemic is substantially higher than what the official sources might suggest. Systematically disadvantaged marginalized subpopulations like migrant workers are coming off worse and bearing a larger weight from the pandemic. Population health was severely and differentially impacted by COVID-19 warranting public health interventions that are sensitive to underlying structural and societal determinants that generate stark inequities and disparities.

## Supplementary Information

**Additional file 1.**

## Data Availability

The datasets generated and/or analyzed during the current study are available in the supplemental material in csv (comma separated values file) format. The World Mortality Dataset is available from this repository; https://github.com/akarlinsky/world_mortality
